# Developing A Mobile App With a Human-Centered Design Lens to Improve Access to Mental Health Care (Mentallys Project): Protocol for an Initial Co-Design Process

**DOI:** 10.2196/47220

**Published:** 2023-08-22

**Authors:** Stéphane Vial, Sana Boudhraâ, Mathieu Dumont, Melanie Tremblay, Sophie Riendeau

**Affiliations:** 1 Centre de Recherche de l'Institut Universitaire en Santé Mentale de Montréal École de Design Université du Québec à Montréal Montréal, QC Canada; 2 Département d’ergothérapie Université du Québec à Trois-Rivières Trois-Rivières, QC Canada; 3 École nationale d'administration publique Québec, QC Canada; 4 Studio Meilleur Monde Montréal, QC Canada

**Keywords:** co-design, human-centered design, e-mental health, design expertise, user engagement, patient-centered design, imaginary prototype

## Abstract

**Background:**

Co-design is one of the human-centered design approaches that allows end users to significantly and positively impact the design of mental health technologies. It is a promising approach to foster user acceptance and engagement in digital mental health solutions. Surprisingly, there is a lack of understanding of what co-design is in this field. In this paper, co-design is approached as a cocreation process involving persons with a lived experience of mental health problems, health professionals, and design experts who lead and facilitate the overall creative process.

**Objective:**

This paper describes an initial co-design research protocol for the development of a mobile app that aims to improve access to mental health care. It highlights the characteristics of a co-design approach in e–mental health rooted in human-centered design and led by design experts alongside health experts. The paper focuses on the first steps (phase 1) of the co-design process of the ongoing Mentallys project.

**Methods:**

This Mentallys project will be located in Montréal (Quebec, Canada). The method approach will be based on the “method stories,” depicting the “making of” this project and reflecting adjustments needed to the protocol throughout the project in specific situations. Phase 1 of the process will focus on the desirability of the app. Targeted participants will include people with a lived experience of mental health problems, peer support workers and clinicians, and 3 facilitators (all design experts or researchers). Web-based sessions will be organized because of the COVID-19 pandemic, using Miro (RealtimeBoard Inc) and Zoom (Zoom Video Communications, Inc). Data collection will be based on the comments, thoughts, and new ideas of participants around the imaginary prototypes. Thematic analysis will be carried out after each session to inform a new version of the prototype.

**Results:**

We conducted 2 stages in phase 1 of the process. During stage 1, we explored ideas through group co-design workshops (divergent thinking). Six co-design workshops were held: 2 with only clinicians (n=7), 2 with peer support workers (n=5) and people with a lived experience of mental health problems (n=2), and 2 with all of them (n=14). A total of 6 facilitators participated in conducting activities in subgroups. During stage 2, ideas were refined through 10 dyad co-design sessions (convergent thinking). Stage 2 involved 3 participants (n=3) and 1 facilitator. Thematic analysis was performed after stage 1, while analytic questioning is being performed for stage 2. Both stages allowed several iterations of the prototypes.

**Conclusions:**

The design of the co-design process, the leadership of the design expertise throughout the process, and the different forms of co-design activities are key elements in this project. We highly recommend that health researchers partner with professional designers or design researchers who are familiar with co-design.

**International Registered Report Identifier (IRRID):**

DERR1-10.2196/47220

## Introduction

### Background

Digital mental health technologies promise greater access to services, improved quality of care, reduced cost, reduced distance and isolation, and enhanced data quality and accessibility [1-3]. However, there are few examples of successful implementation [2], and 2 systematic reviews reveal that few users are using mental health apps for a long period of time [4,5]. One of the reasons for low user engagement is the lack of user perspective in the design of eHealth solutions [6-8]. In the design of sustainable solutions in health care, we should embrace a human-centered design approach at every level of the design process [9].

Human-centered design refers to a wide range of creative approaches that do not belong to engineering design and that allow end users to significantly and positively impact the design of technologies [10,11]. Co-design is one of them [12]. In co-design, end users are not only considered; they also take part in the creation of solutions alongside researchers, designers, and stakeholders [13]. By engaging them in the creative process, co-design can better address end users’ needs and potentially improve user engagement [7,14]. Co-design can also contribute significantly to social change and social innovation [15].

Yet co-design remains an umbrella term, as evidenced by the diversity of initiatives in the scientific literature [16]. There is even a broad range of relationships between actors, from denigration to learning as one [17]. First, it is important to distinguish co-design and participatory design from collaborative design. In collaborative design, participants are “experts operating in their own domain of expertise on a shared problem” [18]. Even if the team is composed of a broad range of expertise, the activities are typically carried out in design teams [19]. The distinction between participatory design and co-design is harder to find in the scientific community, with both terms often being used as synonyms. However, participatory design is a term used in various disciplines to refer to a wide range of participatory activities that are not necessarily related to human-centered design principles and in which there is an irregular and sporadic involvement of end users during the design process. Halskov and Hansen [20] identified fundamental aspects of participatory design: people who are affected by a decision should have an opportunity to influence it; people play critical roles in design by being experts in their own lives; the use situation is the fundamental starting point for the design process; participatory methods are means for users to gain influence in design processes; the goal of participation is to design alternatives to improve quality of life [20]. These aspects also apply to co-design.

In a previous study, we suggested differentiating the 2 terms and reserving the term co-design for a specific form of participatory design rooted in human-centered design [11]. We need to define exactly what it consists of from a research protocol perspective. We consider co-design as a co-creation process at the center of 3 dimensions: collaboration, design, and participation [16]. Potential users are an essential component of any co-design approach, and the quality of the participatory experience offered to them is crucial [14]. Few studies in mental health report using co-design in the development of their applications [11,21], and even fewer embrace its principles [7,22,23]. Most studies in digital mental health simply conduct participatory workshops at specific points during development [24]. Participants usually have the opportunity to provide feedback on the developed solutions, but real cooperation and co-creation remain a challenge [22,25]. Moreover, the role of the expert designers in the development process is often unclear, and in most studies, there is no explicit mention of the presence of designers in the “design” team [11].

However, we have identified a small number of e–mental health studies, still very rare, that assign design and designers an important role in their team, with promising and effective results [26-28]. Co-design requires design. Following the perspective of Manzini, “expert design” (design that comes from people trained in design) is as much a part of a co-design process as “diffuse design” (design that comes from people not trained in design) [29]. The role of the design experts is to design the co-design experience and, as co-design facilitators, to provide conditions conducive to expanding the capabilities of nondesign-trained participants to fully contribute to the co-creation of the solution. People not trained in design bring their natural creativity and experiential knowledge, while design experts bring original ideas and visions, practical design tools, and a structured human-centered design approach derived from their experience [29].

We need to understand what co-design is in e–mental health and how we can implement it. For instance, people not trained in design can be people with a lived experience of mental health problems, families, peer support workers, clinicians, and health professionals. All of them can have their say in the creation process of a digital mental health solution. Therefore, in this paper, we will use this working definition of co-design in e–mental health: a 2-sided human-centered design approach in which (1) end users are engaged from day 1 as partners in the design of the solution and (2) design experts, alongside health researchers, play a key role as facilitators of the overall process and activities.

### Objective

This paper describes an initial co-design research protocol for the development of a mobile app that aims to improve access to mental health care. This protocol highlights the characteristics of a co-design approach in e–mental health rooted in human-centered design and led by design experts alongside health experts. This paper focuses on the first steps of the co-design process for the Mentallys project.

## Methods

### Study Context

#### Methodological Approach and Principles

We want to position this paper in line with the approach of “method stories” in co-design [30,31]. Method stories focus on what the designers actually did with methods in particular circumstances [31]. It focuses not only on the results of co-design sessions but also on the way a method has been used and adjusted in a specific situation [30]. Thus, we will first depict the “making of” the method to be used in this project (see section Study Preparation). In addition, our research protocol for co-design claims a certain flexibility that is directly related to design thinking as a cognitive style [32-35]. Designers alternate between phases of divergent thinking (generating multiple ideas in an exploratory way) and convergent thinking (choosing the best ideas and making choices) [36]. They investigate the problem space more for new possibilities and are more open to a variety of prototyping materials and tools [37]. This leads to adjusting the process several times and adapting it to new creative insights that arise, especially in the early stages. Therefore, the protocol we present in this paper shows the basic structure of our co-design process but, since it is led by design, assumes that adjustments along the way can take place to support flexibility in ideation.

#### Setting

Our study is based on the Mentallys project, located in Montréal (Quebec, Canada). The aim of this project is to improve access to mental health care on a large scale through a mobile app (Mentallys) intended to be distributed for free to the population of a given territory. It is developed from the outset using a co-design process that involves persons with a lived experience of mental health problems, peer support workers, health care professionals (eg, social workers, nurses, psychologists, psychiatrists, and health managers), researchers, design experts, and design students. The Mentallys app will gradually take shape through several steps of co-design, prototyping, and real-world testing in an iterative loop of continuous improvement.

#### Study Preparation

The Mentallys project officially took shape in 2019, prior to the COVID-19 pandemic, and funding and ethics approval were obtained in 2020. Because of the lockdowns and remote work, we had to develop a system of 100% web-based workshops. To this end, we partnered with a design studio specialized in service design and co-design. Due to health restrictions, they had already started this type of practice. One designer (SR) from their team joined us and helped us prepare and initiate stage 1 of the co-design.

First, we set the objective: to explore ideas and desirable features for the Mentallys app. Second, we designed and implemented the co-design experience. The following design experts participated in this step: (1) one social design researcher from another university, (2) one service designer (SR) from the design studio we partnered with, and (3) 5 design researchers and students from our internal co-design team. Their contributions are detailed below.

**Figure 1:**
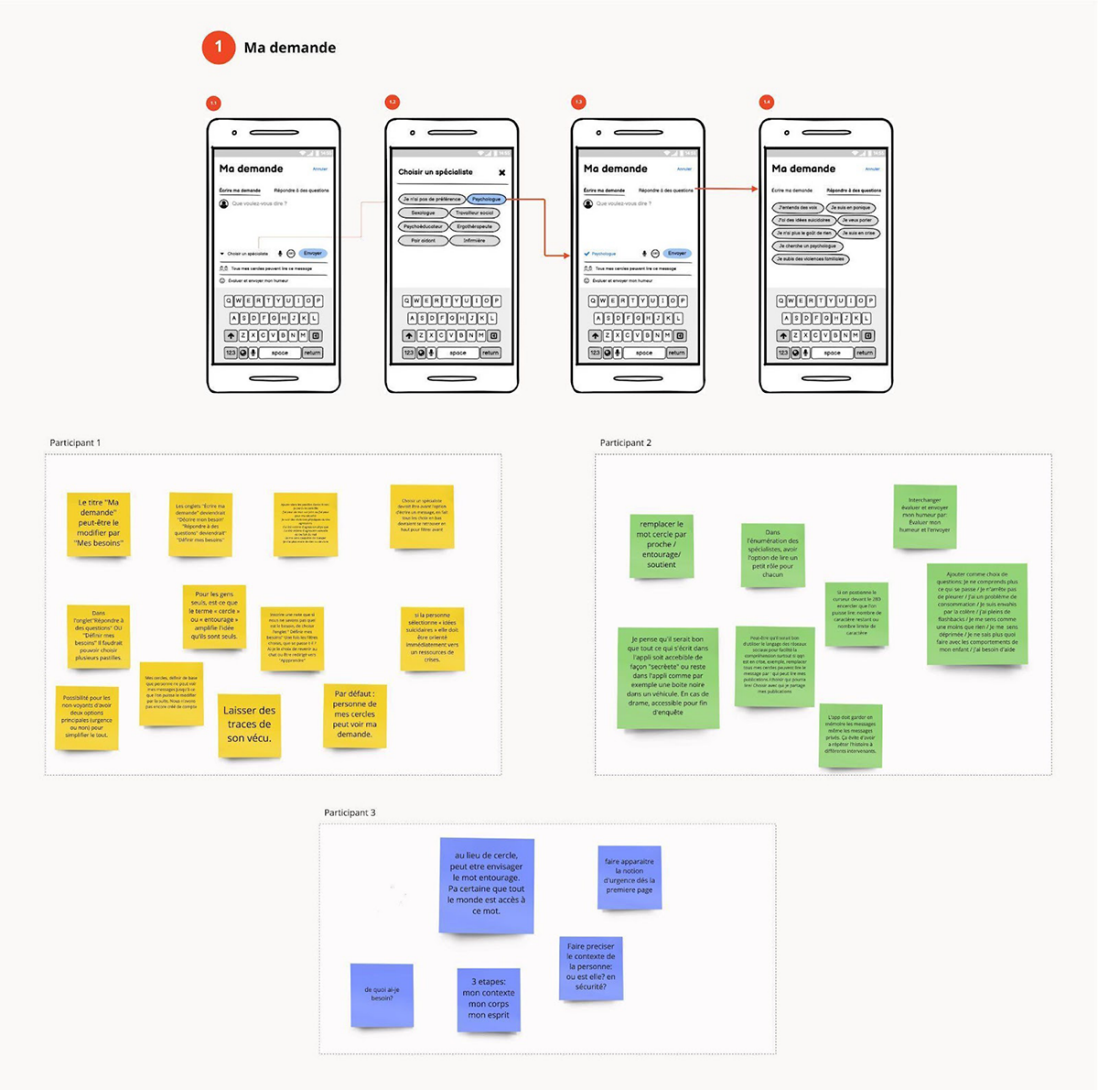
Screenshot of a collaborative space for commenting on an imaginary prototype.

**Figure 2:**
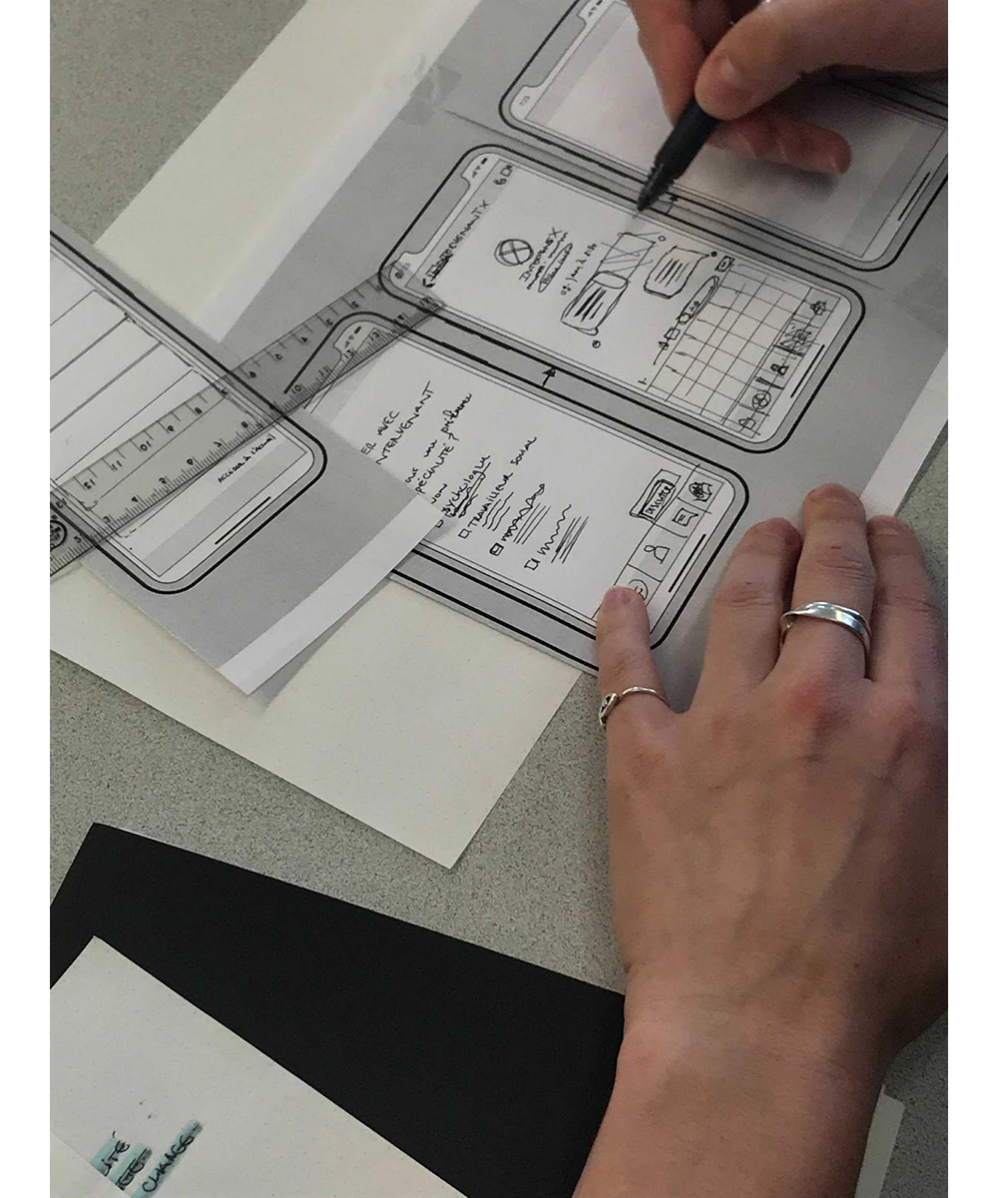
A preliminary sketch of an imaginary prototype in between 2 workshops.

1. The external social design researcher was Prof Philippe Gauthier, who is highly experienced in co-design practice for Montreal public libraries. We conducted an unstructured interview with him to gather his feedback and advice, particularly on how to initiate the co-design process in the most engaging way possible for participants. The interview highlighted the importance of what Dr Gauthier and his colleagues call “imaginary prototypes,” “starting fictions,” or “possible concepts,” which they have shown to play a structuring role in fostering collective ideation, engagement in co-creation, and the organization of co-design workshops [38]. Gauthier and colleagues sometimes refer to these imaginary prototypes as “martyr concepts” to emphasize that they are typically inadequate concepts that participants are invited to criticize [39]. We prefer to think of them as provocative concepts or heuristic provocations, in the sense that they provoke reactions and promote the discovery of new ideas. This led us to build our co-design workshops with imaginary prototypes of the Mentallys app screens, presented as low-fidelity wireframes (having the appearance of being quickly drawn by hand). Workshop participants would then discuss and comment on these imaginary prototypes.
2. The service designer (SR) from the design studio we partnered with is one of the studio’s founders and is the fifth author of this paper. When we contacted her in June 2020, she was already experienced in web-based co-design workshops, which was something relatively new in the early days of the COVID-19 pandemic. With her and thanks to her, we considered a structured co-design process able to take place entirely on the internet via a visual collaboration platform. As part of this process, each co-design workshop will have its own visual dashboard in which collaborative spaces will be organized, allowing each participant to comment on an imaginary prototype using web-based post-it notes (Figure 1). The process will rely on the role of several design experts acting as facilitators of co-creation with the participants. These designer-facilitators will act on several levels: stimulating individual and collective ideation, inspiring and moderating the conversation, supporting the visual formatting of ideas, and helping with the use of the software. Our partner service designer (SR) will participate as a cofacilitator in the first 2 co-design workshops to help us fine-tune the method, and our internal co-design team will then conduct the rest of the workshops alone.
3. Our internal co-design team was initially composed of 3 people (SV, SB, and SR) and then evolved to 5 people to meet the expected needs for facilitation (4 facilitators on average per workshop): 1 design researcher (SV) experienced in interaction design with a background in clinical psychology and philosophy (principal investigator); 1 postdoctoral researcher (SB) with a background in industrial design and research expertise in co-design; 1 researcher in occupational therapy (MD) familiar with new technologies and participatory design; 1 doctoral student in service design with expertise in interaction design; and 1 master’s student in design with expertise in graphic design and with personal experience of mental health problems. Highly experienced in design and familiar with the subject of mental health, our team will adopt the co-design method from the first workshops and then develop, adapt, and customize it through subsequent workshops. This will include the continuous design work of the co-design experience between workshops (Figure 2).

### Study Design

Our study is based on the core concepts of design thinking. Originally described as a cognitive style [33,34,40], design thinking is now widely recognized as an organizational resource for innovation [41]. It was popularized by American design agency IDEO, especially its former CEO Tim Brown, and defined as a methodology for innovation with a human-centered design ethos [42]. Our study design is inspired by design thinking as integrating thinking composed of 3 dimensions: desirability, feasibility, and viability [43]. Desirability relates to “what is desirable from a human point of view,” that is, people’s needs; feasibility relates to “what is technologically feasible,” and viability relates to “what is economically viable” [44].

In an e–mental health context, we assume that (1) desirability relates to what is desirable and acceptable for people with a lived experience of mental health problems, families, peer support workers, clinicians, and health professionals, which takes into account their needs, expectations, frustrations, and concerns; (2) feasibility relates to what is digitally and organizationally feasible within the health system; and (3) viability relates to what is economically acceptable for all stakeholders (business model) and environmentally sustainable.

Based on these 3 dimensions, we are planning 3 design phases: phase 1, focusing on exploring desirability, phase 2 exploring feasibility, and phase 3 exploring viability. In this study, we present only phase 1, desirability, since it will be entirely focused on co-design.

### Recruitment

After obtaining an ethical certificate for the Mentallys project, we will launch a call for participation through our channels for the 3 types of participants: persons with a lived experience of mental health problems (n=10), peer support workers (n=10), and health professionals (n=10). Eligibility criteria in terms of age and language will be the same for all participants: Age (18-65 years) and being able to communicate in French. A diverse representation of ages, genders, and ethnicities will be sought.

For those with lived experience, additional eligibility criteria will be added: having experienced at least one anxiety or depressive episode in adulthood and within the past 2 years that has fully resolved or is currently at the mild to moderate level.

### Ethics Approval

This project has been approved by the Institutional Human Research Ethics Committee of the Université du Québec à Montréal (No. 4425_e_2020).

Participants will sign an information and consent form, giving their free and informed consent to the research and publication, including their participation. They will be well informed that all personal information collected about them during the study will be coded to ensure confidentiality. This data will be kept under lock and key by the study leader for a period of 5 years after the end of the validity period of the research grant obtained, after which it will be destroyed. During this period, only members of the research team will have access to them. If the results of this research are presented or published, they will not be identified. All participants will receive monetary compensation (ranging from US $55 to US $75) for each time they participate.

### Data Collection and Analysis

Textbox 1 shows the initial data collection and analysis strategy.

**Figure 3:**
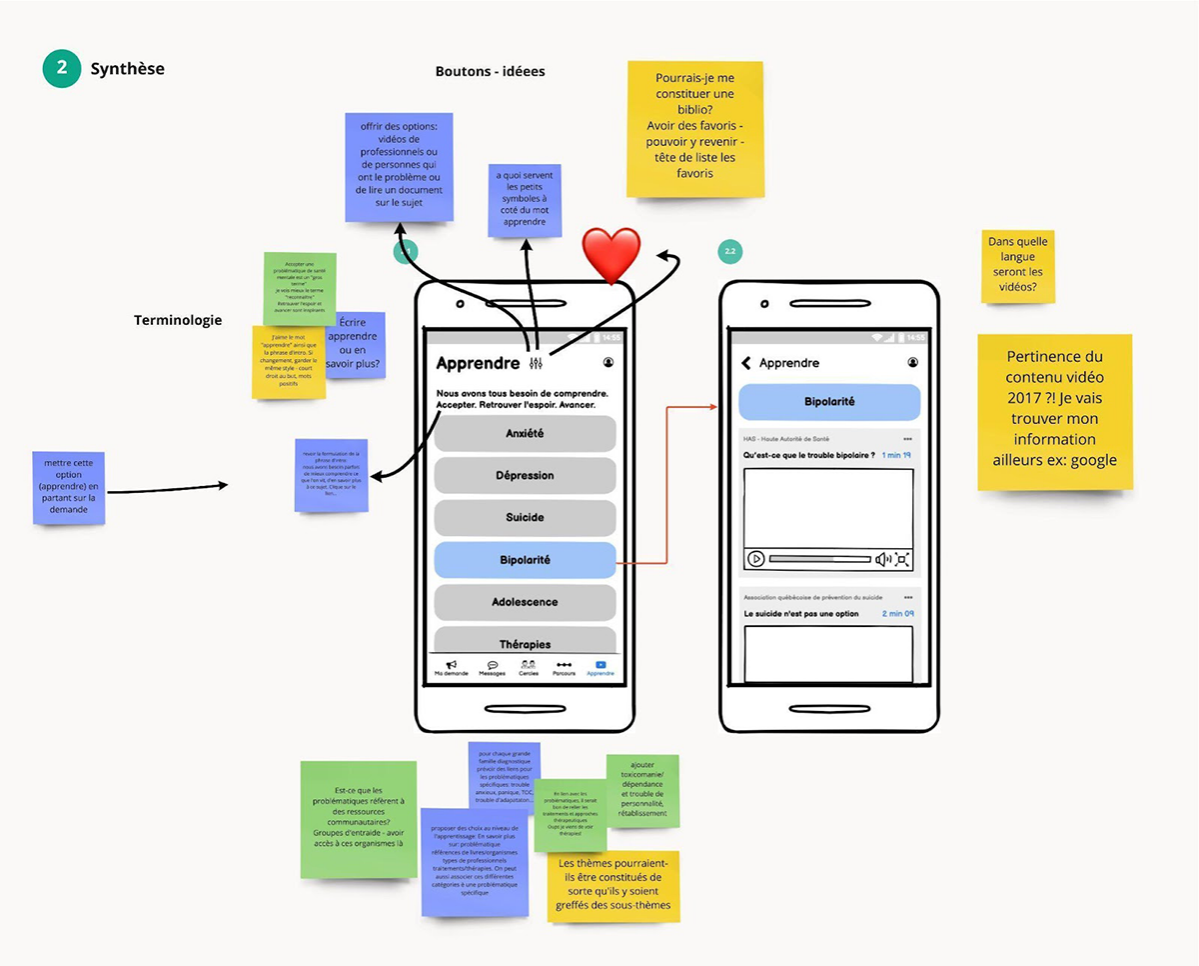
Sample of low-fidelity wireframes used for imaginary prototypes.

Participants will be divided into subgroups of 4 or 5 participants (1 subgroup per facilitator) and led by the facilitators to comment freely, generate thoughts, and share new ideas around the imaginary prototypes while being asked reflective questions by the facilitator. Imaginary prototypes will be delivered to participants in the form of wireframes. Well-known in the web industry, wireframes are visual schematics or screen blueprints that help represent and communicate the content and features of an app or website. Most often, wireframes are made with a low-fidelity design, that is, imitating a quick drawing by hand or a simple design with minimal graphic details (Figure 3).

**Figure 4:**
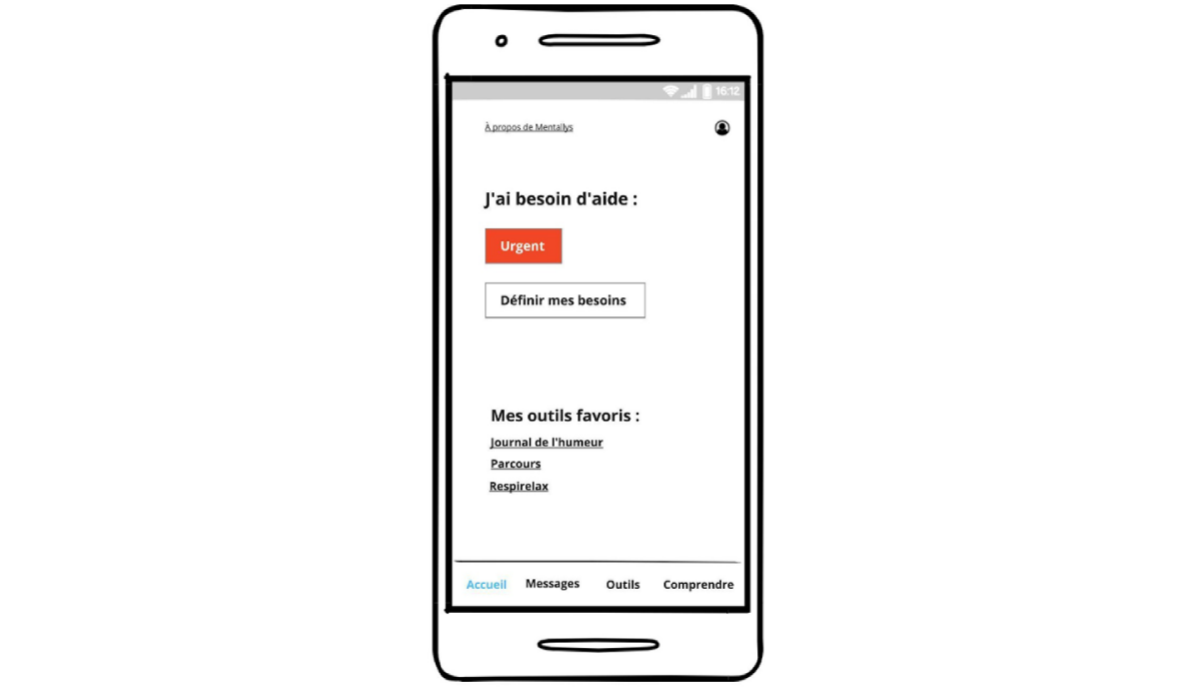
Sample of a wireframe incorporating participants’ ideas created in real time during a workshop.

Participants will directly comment on wireframes through the collaborative visual platform, mostly using web-based post-its (a different color for each participant; Figure 3). If necessary, a designer-facilitator with expertise in interaction design will be able to create a wireframe in real time based on the ideas shared by the participants using other annotation tools (Figure 4).

**Plan for the Mentallys co-design process (phase 1).**

Dimension

- Desirability

Objective

- Exploring ideas and desirable features for the Mentallys app

Nature of activities

- Group co-design workshops

Number of activities

- 5

Expected duration

- 3 months

Participants

- 10 clinicians
- 10 people with lived experience of a mental health problem
- 10 peer support workers

Facilitators

- 1 service designer from a partner design studio (SR)
- 1 design researcher experienced in interaction design (SV)
- 1 postdoctoral researcher in design and co-design (SB)

Modality

- Online

Data to be collected

- Participant verbatim comments on prototypes (audio data)
- Participant verbatim discussions (audio data)
- Web-based post-its and comments on prototypes (visual or textual data)

Data collection tools

- Video conferencing software (Zoom; Zoom Video Communications, Inc)
- Visual collaboration platform (Miro; RealtimeBoard Inc)

Data analysis

- Thematic analysis [45]

All the collected data will be analyzed through a thematic analysis. Thematic analysis is essentially a method for identifying and synthesizing the themes present in a corpus. In accordance with Paillé and Mucchielli [45], themes will be assigned to ideas, questions, and challenges that emerge from the workshop. They will then be grouped into categories. The process will be conducted jointly by the second author (SB) and a research assistant (a doctoral student) familiar with this process. The differences will be discussed between analysts in order to reach an interrater agreement.

## Results

### Overview

Our study received funding on June 2, 2020, from the Fonds de recherche du Québec (#2021-AUDC-283359). It received ethical approval on December 1, 2020, from the “Comité institutionnel d’éthique de la recherche avec des êtres humains” at the Université du Québec à Montréal (certificate number: 4425_e_2020). Recruitment began immediately, in December 2020, and lasted until the end of January 2021.

Here, we present the status of the co-design process for the Mentallys project. Consistent with the flexible methodology principle, the co-design activities have been conducted in 2 stages so far: one stage in group co-design workshops (February 2021 to June 2021) and one stage in dyad co-design sessions (April 2022 to July 2022). Only some of the participants from stage 1 participated in stage 2, since stage 2 required a very small number of people (Table 1).

**Table 1:** Sociodemographic data of the participants in both stages.

Variable			People with a lived experience of a mental health problem (n=2)		Peer support workers^a^ (n=5)	Clinicians (n=7)
**Gender, n (%)**						
	Women	2 (100)		4 (80)		4 (57)
	Men	0 (0)		1 (20)		3 (43)
**Age range (years), n (%)**						
	18-24	0 (0)		0 (0)		0 (0)
	25-34	0 (0)		0 (0)		0 (0)
	35-44	1 (50)		0 (0)		1 (14)
	45-54	1 (50)		4 (80)		2 (28)
	55-65	0 (0)		1 (20)		4 (58)
**Geographical distribution, n (%)**						
	Montréal	1 (50)		2 (40)		4 (58)
	Other regions in Quebec	1 (50)		3 (60)		1 (14)
	France	0 (0)		0 (0)		2 (28)

^a^Peer support workers are also people with a lived experience of a mental health problem.

### Stage 1: Exploring Ideas Through Group Co-Design Workshops

Stage 1 took place from February 2021 to June 21, 2021. We recruited 14 end users (see table 1). Participants have been divided into 2 groups. Group A included 7 clinicians (5 psychologists and 2 psychiatrists), and group B included 7 people with lived experience of a mental health disorder and peer support workers. We conducted 6 co-design workshops (rather than 5, as planned). Two workshops were held with group A, 2 workshops were held with group B, and 2 workshops were held simultaneously with both groups (Textbox 2).

**Results summary of co-design (stage 1).**

Thinking

- Divergent

Dimension

- Desirability

Objective

- Exploring ideas and desirable features for the Mentallys app
- Collecting user experiences and issues

Nature of activities

- Group co-design workshops

Number of activities

- 6

Duration

- 5 months

Data collection period

- February 2021 to June 2021

Participants

- 7 clinicians (5 psychologists, 2 psychiatrists)
- 2 people with lived experience of a mental health problem
- 5 peer support workers

Facilitators

- 1 service designer from a partner design studio (SR)
- 1 design researcher experienced in interaction design (SV)
- 1 postdoctoral researcher in design and co-design (SB)
- 1 researcher in occupational therapy experienced in participatory design (MD)
- 1 PhD student in service design with a background in interaction design
- 1 master’s student in design with lived experience of a mental health problem

Modality

- Online

Prototypes (medium)

- Imaginary (wireframes)

Data types

- Participant verbatim comments on prototypes (audio data)
- Participant verbatim discussions (audio data)
- Web-based post-its and comments on prototypes (visual or textual data)
- Additional prototypes made in real time by facilitators (images)
- Ad hoc diagrams and visual maps (images)

Data collection tools

- Video conferencing software (Zoom; Zoom Video Communications, Inc)
- Visual collaboration platform (Miro; RealtimeBoard Inc)

Data analysis

- Thematic analysis [45]

This stage encouraged divergent thinking with the goal of generating ideas and identifying desirable features for the Mentallys app through imaginary prototypes and social conversations with different types of end users. Six facilitators were involved in the co-design activities, as it turned out that small groups were more appropriate for web-based workshops.

**Figure 5:**
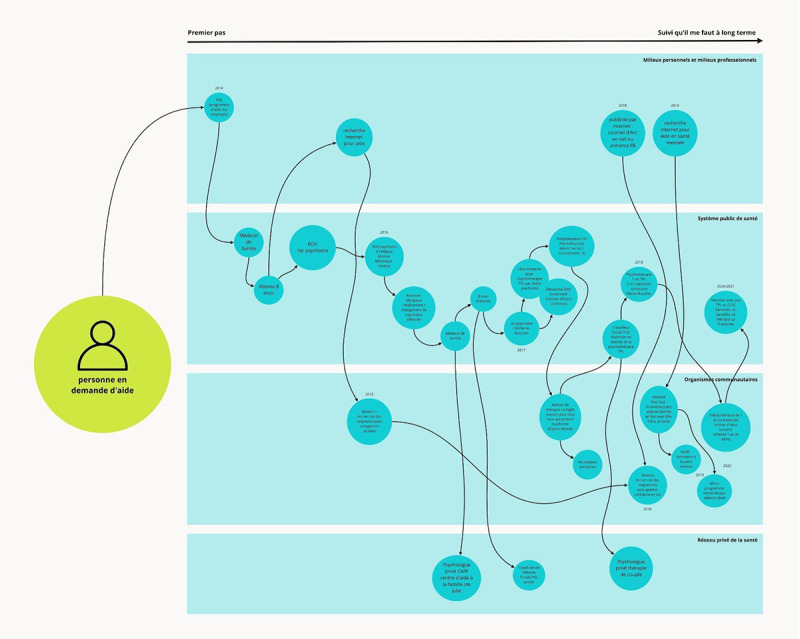
Sample of a care pathway map.

As planned, audio feedback from participants on the prototypes was collected and analyzed using thematic analysis. Moreover, visual data were eventually collected during a workshop with group B as we realized that we did not sufficiently understand their reality, which hindered the co-design process. Participants were invited to create care pathway maps (Figure 5), adjusting the initial protocol according to our flexible methodological approach. Those data have been analyzed using qualitative and quantitative content analysis [46].

### Stage 2: Refining Ideas Through Dyad Co-Design Sessions

Stage 2 ran from April to July 2022. Two participants continued their involvement (1 peer support worker and 1 person with a lived experience of a mental disorder), and 1 health professional was recruited (a medical resident). The co-design activities did not take the usual form of group workshops but were rather conducted as dyad sessions, again adjusting for specific situations according to our flexible methodological approach (Textbox 3). This was necessary in order to explore sensitive and confidential topics while respecting the privacy of the participants and building trust. A total of 10 dyad co-design sessions were conducted for 3 months: 5 web-based sessions with the person with lived experience of a mental health problem, 2 web-based sessions with the peer support worker, and 3 in-person sessions with the medical resident.

**Results summary of co-design (stage 2).**

Thinking

- Convergent

Dimension

- Desirability

Objective

- Making choices among possible features
- Refining and validating key features of the Mentallys app

Nature of activities

- Dyad co-design sessions

Number of activities

- 7

Duration

- 3 months

Data collection period

- April 2022 to July 2022

Participants

- 1 clinician
- 1 person with lived experience of a mental health problem
- 1 peer support worker

Facilitators

- 1 design researcher (principal investigator)

Modality

- Online

Prototypes (medium)

- Real (graphic, interactive, clickable)

Data types

- Participant verbatim comments on prototypes (audio data)
- Participant verbatim discussions (audio data)
- Facial expressions and body language (video data)
- Web-based post-its and comments on prototypes (visual or textual data)
- Additional prototypes made in real time by facilitators (images)
- Ad hoc diagrams and visual maps

Data collection tools

- Video conferencing software (Zoom; Zoom Video Communications, Inc)
- User Interface (UI) design prototyping software (Figma; Figma, Inc)
- Research diary

Data analysis

- Analytic questioning [45]

**Figure 6:**
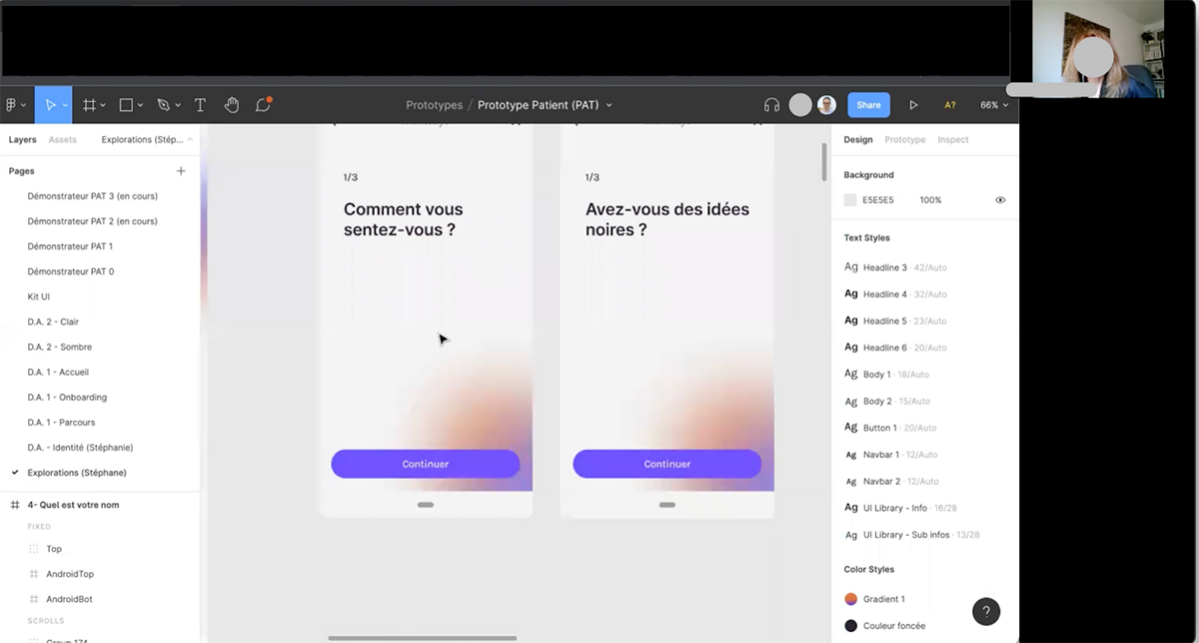
High-fidelity prototype being commented on and improved in real time through screen sharing.

**Figure 7:**
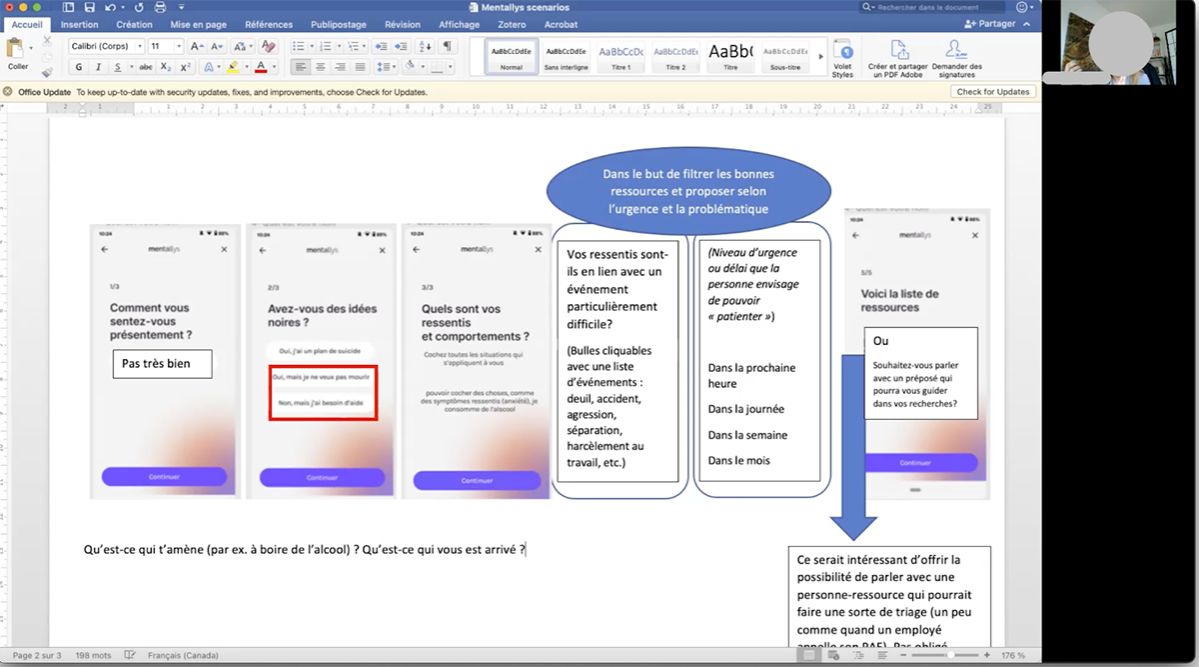
Mix of high-fidelity prototypes and new ideas generated by the participant in a Word document.

This stage encouraged convergent thinking with the goal of validating ideas that can satisfy end users in the real world through real prototypes. One facilitator (SV) was involved in all sessions. For each session, the objective was to refine and validate a set of features through real, clickable, and testable prototypes shared on screen using a collaborative design and prototyping tool. End users could either verbally comment on the features or suggest new features in real time (Figure 6) or think about it at home and bring ideas to the next session (Figure 7).

Audio feedback from participants on the prototypes was collected and is being analyzed using an analytic questioning method inspired by Paillé and Mucchielli [45]. In this method, research questions are further developed and used as a framework to analyze the data. This is the most suitable approach when answers to a certain number of explicit questions are sought [45], which is the case for this convergent stage.

## Discussion

### Principal Findings

In this study, we described the characteristics of a co-design research protocol in e–mental health rooted in human-centered design and led by design experts alongside health experts. This protocol focused on initializing the co-design process and centered on exploring the desirability of a mobile app that aims to improve access to mental health care by collecting data on the needs, expectations, frustrations, and concerns of people with lived experience of mental health problems, peer support workers, and clinicians. We successfully implemented a 2-stage protocol, with group co-design workshops in the first stage and dyad co-design sessions in the second. For both stages, the role of expert design facilitators was crucial. In this discussion, we want to highlight a few challenges and lessons learned.

### Initiating the Co-Design

Our preliminary design work on the co-design experience was critical to the implementation and success of the process, especially partnering with a design studio specialized in service design to help us structure the approach and become familiar with the best-suited tools. This allowed us to prepare and conduct the first workshops according to the best practices in human-centered design and, afterward, to continue doing so independently. We now have the full capacity to devise and manage further co-design stages thanks to the skills our team has acquired. These skills are necessary to create a positive group dynamic that encourages each participant’s creativity. In co-design, co-designers participate with different perspectives, which can lead to frustration and conflicts among participants who do not always understand each other [47-49]. By structuring activities using a human-centered design perspective, optimizing collaboration by orienting the process toward positive and constructive interactions among participants, and leading participants into a creative design thinking mode, facilitators can create this positive dynamic and foster creativity [14]. Expert design skills are also needed to ground the co-design process in concrete prototypes from the start, which become mediators of conversation and ideation. Prototypes are a useful co-design tool to engage participants in the design conversation. Prototypes allow opposite opinions to exist and are socio-material, welcoming a neutral space and facilitating the negotiation of issues [50]. In this project, where the subjects discussed were often very sensitive, prototypes played a significant mediator role to ensure constructive conversation.

### Adapting the Process Along the Way

Even when highly organized and structured, co-design is a creative process that inevitably involves unforeseen ideas and insights. Unique to each design process, this characteristic is known as the “creative leap” [51]. To facilitate a co-design process, one must be attentive to these creative leaps, be able to welcome them, and integrate them into the process along the way. This involves moving away from the rigidity of traditional methodological protocols in research in order to be able to adapt the protocol as it unfolds. The difficulty lies in maintaining a balance between following the planned protocol and making the necessary adjustments resulting from creative leaps. In this study, creative leaps led us to focus first on 1 major phase (desirability) instead of tackling all 3 phases from the outset (desirability, feasibility, and viability), which were itself divided into 2 stages (group co-design and dyad co-design). Therefore, we could only study the desirability dimension, as it turned out to require much more time. Indeed, rather than the 3 months planned, this study lasted a total of 8 months (5 months for stage 1 and 3 months for stage 2). Other similar studies also mentioned that some stages of the co-design process took longer than expected [52].

### Doing Co-Design in Dyads

Our study suggests that co-design can be dyadic. During stage 2, we organized the co-design sessions in dyads, that is, one-to-one meetings, instead of the standard group workshops. Even if it was not a group activity, it was true collective creativity. Since co-design is a collaboration between diffuse design and expert design [29], it can start with 2 people, provided one of them is a design expert. As a specific point of co-creation, co-design is collective creativity that can be shared “by two or more people” [53]. In our protocol, each dyad session was a conversation between a person not trained in design (the end user) and a member of our team with design expertise (the principal investigator). Both were equal speakers, each able to bring his or her points to the table. The 3 main aspects of co-design, that is, collaboration, design, and participation [16], were present but manifested dyadically. Through the accumulation of dyadic sessions, a mass effect in data collection occurs that is comparable to that of a larger group. In-depth analysis of the results will provide a better understanding of the contribution of dyadic sessions to the design process.

### Leading Co-Design Thanks to Design Expertise

Our co-design research protocol focused on a close and effective collaboration between experts in design and end users throughout the process. While other e–mental health research projects have shown less involvement on the part of the designers in their process, we aimed at creating a continuous collaboration environment between designers and users. In fact, very few studies mention the presence of designers on their teams and mostly focus on involving end users [11]. The challenge in these types of projects is how to properly incorporate users’ needs into the project, redefine them properly, and bridge them while the project evolves. Design expertise leadership is a key component in achieving that goal since design practice and research focus on users’ unmet needs through well-understood and applied inclusive design approaches coming from design expertise [54]. Designers are sensitive to how care is received through user-centered practices [55] and can use a variety of flexible methods and tools to enhance communication about the project.

### Limitations and Future Work

While co-design is central to our approach, we recognize that other elements must be taken into account to ensure the development of a mental health app that meets the needs of users and is likely to be adopted. Close collaboration with the software development team is important, as is usability testing of the app [56] or user experience design [57]. One should also assess the viability of the solution, develop a business model for the app, and plan the implementation of the technology [42,58]. This points to the importance of transdisciplinary work in the development of e–mental health solutions from a human-centered design perspective [59]. This is why the Mentallys project relies on other research and development activities complementary to co-design: a full design team, including branding design experts and user experience and user interface designers; a business accelerator program and business model definition coaching; a partner technology company that cosupervises the software development; and several partner health institutions to conduct real-world testing.

### Conclusions

This paper presented a research protocol rooted in human-centered design related to the Mentallys project. More precisely, we sought to present how a co-design approach can be used to initiate the design and development of a mental health mobile app. The preliminary design of the co-design process, the leadership of the design expertise throughout the process, and the different forms of co-design activities are key elements in this project. We expect that this process can lead to the development of an app that is more engaging and better meets the important needs of people seeking access to mental health services. Overall, this research protocol may inspire other research teams that want to integrate co-design as a more user-centered approach to the development of e–mental health solutions. For this purpose, we highly recommend that health researchers partner with professional designers or design researchers who are familiar with co-design.

## Data Availability

The data sets generated during this study are not publicly available since the research is still ongoing, but they are available from the corresponding author on reasonable request.

## References

[1] Lal S (2019). E-mental health: promising advancements in policy, research, and practice. Healthc Manage Forum.

[2] Mohr DC, Lyon AR, Lattie EG, Reddy M, Schueller SM (2017). Accelerating digital mental health research from early design and creation to successful implementation and sustainment. J Med Internet Res.

[3] Torous J, Myrick KJ, Rauseo-Ricupero N, Firth J (2020). Digital mental health and COVID-19: using technology today to accelerate the curve on access and quality tomorrow. JMIR Ment Health.

[4] Amagai S, Pila S, Kaat AJ, Nowinski CJ, Gershon RC (2022). Challenges in participant engagement and retention using mobile health apps: literature review. J Med Internet Res.

[5] Baumel A, Muench F, Edan S, Kane JM (2019). Objective user engagement with mental health apps: systematic search and panel-based usage analysis. J Med Internet Res.

[6] Birnbaum F, Lewis D, Rosen RK, Ranney ML (2015). Patient engagement and the design of digital health. Acad Emerg Med.

[7] Thabrew H, Fleming T, Hetrick S, Merry S (2018). Co-design of eHealth interventions with children and young people. Front Psychiatry.

[8] Torous J, Nicholas J, Larsen ME, Firth J, Christensen H (2018). Clinical review of user engagement with mental health smartphone apps: evidence, theory and improvements. Evid Based Ment Health.

[9] Searl MM, Borgi L, Chemali Z (2010). It is time to talk about people: a human-centered healthcare system. Health Res Policy Syst.

[10] Norman DA (1988). The Psychology of Everyday Things.

[11] Vial S, Boudhraâ S, Dumont M (2022). Human-centered design approaches in digital mental health interventions: exploratory mapping review. JMIR Ment Health.

[12] Steen M (2011). Tensions in human-centred design. CoDesign.

[13] Sanders EBN, Stappers PJ (2008). Co-creation and the new landscapes of design. CoDesign.

[14] Tremblay M, Hamel C, Viau-Guay A, Giroux D (2022). User experience of the co-design research approach in eHealth: activity analysis with the course-of-action framework. JMIR Hum Factors.

[15] Mahoney A, Li I, Haskelberg H, Millard M, Newby JM (2021). The uptake and effectiveness of online cognitive behaviour therapy for symptoms of anxiety and depression during COVID-19. J Affect Disord.

[16] Tremblay M (2022). Université Laval. Perspectives sur l'approche de codesign: Analyse de l'expérience des participants dans un projet de codesign en cybersanté.

[17] Harder MK, Burford G, Hoover E (2013). What is participation? Design leads the way to a cross-disciplinary framework. Des Issues.

[18] Kvan T (2000). Collaborative design: what is it?. Automation in Construction.

[19] Schober M, Erlhoff M, Marshall T (2008). Collaborative design. Design Dictionary: Perspectives on Design Terminology.

[20] Halskov K, Hansen NB (2015). The diversity of participatory design research practice at PDC 2002–2012. Int J Hum-Comput Stud.

[21] Bergin AD, Vallejos EP, Davies EB, Daley D, Ford T, Harold G, Hetrick S, Kidner M, Long Y, Merry S, Morriss R, Sayal K, Sonuga-Barke E, Robinson J, Torous J, Hollis C (2020). Preventive digital mental health interventions for children and young people: a review of the design and reporting of research. NPJ Digit Med.

[22] Hodson E, Dadashi N, Delgado R, Chisholm C, Sgrignoli R, Swaine R (2019). Co-design in mental health; Mellow: a self-help holistic crisis planning mobile application by youth, for youth. The Design Journal.

[23] Nakarada-Kordic I, Hayes N, Reay SD, Corbet C, Chan A (2017). Co-designing for mental health: creative methods to engage young people experiencing psychosis. Des Health.

[24] Jones RB, Stallard P, Agha SS, Rice S, Werner-Seidler A, Stasiak K, Kahn J, Simpson SA, Alvarez-Jimenez M, Rice F, Evans R, Merry S (2020). Practitioner review: co-design of digital mental health technologies with children and young people. J Child Psychol Psychiatry.

[25] Buus N, Juel A, Haskelberg H, Frandsen H, Larsen JLS, River J, Andreasson K, Nordentoft M, Davenport T, Erlangsen A (2019). User involvement in developing the MYPLAN mobile phone safety plan app for people in suicidal crisis: case study. JMIR Ment Health.

[26] Garety P, Ward T, Emsley R, Greenwood K, Freeman D, Fowler D, Kuipers E, Bebbington P, Rus-Calafell M, McGourty A, Sacadura C, Collett N, James K, Hardy A (2021). Effects of SlowMo, a blended digital therapy targeting reasoning, on paranoia among people with psychosis: a randomized clinical trial. JAMA Psychiatry.

[27] Hardy A, Wojdecka A, West J, Matthews E, Golby C, Ward T, Lopez ND, Freeman D, Waller H, Kuipers E, Bebbington P, Fowler D, Emsley R, Dunn G, Garety P (2018). How inclusive, user-centered design research can improve psychological therapies for psychosis: development of SlowMo. JMIR Ment Health.

[28] Jongeneel A, Scheffers D, Tromp N, Nuij C, Delespaul P, Riper H, van der Gaag M, van den Berg D (2018). Reducing distress and improving social functioning in daily life in people with auditory verbal hallucinations: study protocol for the 'Temstem' randomised controlled trial. BMJ Open.

[29] Manzini E (2015). Design, When Everybody Designs: An Introduction to Design for Social Innovation.

[30] Hendriks N, Slegers K, Duysburgh P (2015). Codesign with people living with cognitive or sensory impairments: a case for method stories and uniqueness. CoDesign.

[31] Lee JJ (2014). The true benefits of designing design methods. Artifact.

[32] Archer B (1979). Design as a discipline. Des Stu.

[33] Cross N (1982). Designerly ways of knowing. Des Stud.

[34] Cross N (2006). Designerly Ways of Knowing.

[35] Dorst K, Dijkhuis J (1995). Comparing paradigms for describing design activity. Des Stu.

[36] Cross N (2008). Engineering Design Methods. 4th Edition.

[37] Yu F, Pasinelli M, Brem A (2017). Prototyping in theory and in practice: a study of the similarities and differences between engineers and designers. Creativity Innov Manag.

[38] Abrassart C, Gauthier P, Proulx S, Martel MD (2015). Le design social: une sociologie des associations par le design? Le cas de deux démarches de codesign dans des projets de rénovation des bibliothèques de la ville de Montréal. Lien social et Politiques.

[39] Levée V (2019). Codesign: La parole aux citoyens. Ordre des architectes du Québec.

[40] Dorst K (2011). The core of 'design thinking' and its application. Des Stu.

[41] Kimbell L (2015). Rethinking design thinking: part i. Des Cult.

[42] Brown T (2008). Design thinking. Harvard Business Review.

[43] Brown T, Katz B (2009). Change by Design: How Design Thinking Transforms Organizations and Inspires Innovation (1st Edition).

[44] (2018). Design Thinking defined. IDEO Design Thinking.

[45] Paillé P, Mucchielli A (2012). A. Colin. L'analyse qualitative en sciences humaines et sociales.

[46] Morgan DL (1993). Qualitative content analysis: a guide to paths not taken. Qual Health Res.

[47] Bossen C, Dindler C, Iversen OS (2012). Impediments to user gains: experiences from a critical participatory design project. Proc Participatory Des Conf - PDC'12.

[48] Bowen S, McSeveny K, Lockley E, Wolstenholme D, Cobb M, Dearden A (2013). How was it for you? Experiences of participatory design in the UK health service. CoDesign.

[49] Zowghi D, da Rimini F, Bano M (2015). Problems and challenges of user involvement in software development: an empirical study. https://dl.acm.org/doi/proceedings/10.1145/2745802.

[50] Tatro C, Fleming J (2017). Generative design research: using metaphor to capture complexity. Proc Int Symp Human Factors Ergon Health Care.

[51] Cross N (1997). Descriptive models of creative design: application to an example. Des Stud.

[52] Tremblay M, Latulippe K, Mc Giguere A, Provencher V, Poulin V, Dubé V, Guay M, Ethier S, Sévigny A, Carignan M, Giroux D (2019). Requirements for an electronic health tool to support the process of help seeking by caregivers of functionally impaired older adults: co-design approach. JMIR Aging.

[53] Sanders EBN, Stappers PJ (2020). Convivial Toolbox: Generative Research for the Front End of Design (5th Printing 2020).

[54] Rivard L, Lehoux P, Hagemeister N (2021). Articulating care and responsibility in design: a study on the reasoning processes guiding health innovators' 'care-making' practices. Des Stu.

[55] Ku B, Lupton E, Ku B (2020). Health Design Thinking: Creating Products and Services for Better Health.

[56] Inal Y, Wake JD, Guribye F, Nordgreen T (2020). Usability evaluations of mobile mental health technologies: systematic review. J Med Internet Res.

[57] Garrett JJ, Riders N (2011). The Elements of User Experience.

[58] van Gemert-Pijnen JEWC, Nijland N, van Limburg M, Ossebaard HC, Kelders SM, Eysenbach G, Seydel ER (2011). A holistic framework to improve the uptake and impact of eHealth technologies. J Med Internet Res.

[59] Vial S, Boudhraâ S (2022). Design for e-Mental health: toward a new health intervention research approach. Revolutions in Product Design for Healthcare.

